# Long-term cisplatin nephrotoxicity after childhood cancer: a systematic review and meta-analysis

**DOI:** 10.1007/s00467-023-06149-9

**Published:** 2023-09-20

**Authors:** Jessica Schofield, Matthew Harcus, Barry Pizer, Andrea Jorgensen, Stephen McWilliam

**Affiliations:** 1 Department of Women’s and Children’s Health, University of Liverpool, Liverpool, UK; 2 Department of Paediatric Oncology, Alder Hey Children’s Hospital, Liverpool, UK; 3 Department of Biostatistics, University of Liverpool, Liverpool, UK

**Keywords:** Cisplatin, Hypomagnesaemia, GFR, Nephrotoxicity

## Abstract

**Background:**

Cisplatin is a chemotherapeutic drug commonly used in the treatment of many childhood solid malignancies. It is known to cause long-term nephrotoxicity, most commonly manifesting as reduced glomerular filtration rate and hypomagnesaemia. Existing literature regarding the epidemiology of long-term nephrotoxicity in childhood cancer describes large variation in prevalence and risk factors.

**Objectives:**

This study is to evaluate the prevalence of, and risk factors for, long-term cisplatin nephrotoxicity after treatment for childhood cancer.

**Study eligibility criteria:**

Studies were eligible for inclusion if they: (i) evaluated participants treated with cisplatin who were diagnosed with cancer < 18 years of age; (ii) investigated any author-defined measure of nephrotoxicity; and (iii) performed this evaluation 3 or more months after cisplatin cessation. Studies whose scope was broader than this were included if appropriate subgroup analysis was performed.

**Results:**

Prevalence of reduced glomerular filtration rate (GFR) ranged between 5.9 and 48.1%. Pooled prevalence of reduced GFR using studies with a modern consensus threshold of 90 ml/min/1.73 m^2^ was 29% (95% CI 0.0–58%). Prevalence of hypomagnesaemia ranged between 8.0 and 71.4%. Pooled prevalence of hypomagnesaemia was 37% (95% CI 22–51%). Substantial heterogeneity was present, with *I*^2^ statistics of 94% and 73% for reduced GFR and hypomagnesaemia respectively. All large, long-term follow-up studies described increased risk of reduced GFR with increasing cumulative cisplatin dose. Included studies varied as to whether cisplatin was a risk factor for proteinuria, and whether age was a risk factor for cisplatin nephrotoxicity.

**Limitations:**

A wide range of study methodologies were noted which impeded analysis. No studies yielded data from developing health-care settings. No non-English studies were included, further limiting generalisability.

**Conclusions:**

Both of the most common manifestations of long-term cisplatin nephrotoxicity have a prevalence of approximately a third, with increasing cumulative dose conferring increased risk of nephrotoxicity. Further work is needed to characterise the relationship between reduced GFR and hypomagnesaemia, investigate other risk factors and understand the interindividual variation in susceptibility to nephrotoxicity.

**Supplementary Information:**

The online version contains supplementary material available at 10.1007/s00467-023-06149-9.

## Introduction

Renal injury is a common problem in paediatric oncology. It can result from direct malignant invasion by urinary system cancers and their subsequent excision, nephrotoxic chemotherapy, irradiation and issues such as tumour lysis syndrome and sepsis [1]. Such injury has long-term consequences for childhood cancer survivors (CCS). A retrospective cohort study of over 10,000 CCS in North America demonstrated a ninefold increased risk of developing kidney failure compared to their siblings [2].

Cisplatin is a platinum-based chemotherapeutic drug which is commonly used in the treatment of paediatric solid tumours such as osteosarcoma, neuroblastoma, hepatoblastoma and various brain tumours [3]. Listed as an essential medicine for children by the World Health Organisation [4], it is likely to remain a key element of children’s cancer therapy for the foreseeable future and recent interest in delivering cisplatin prodrugs via nanoparticles may optimise its therapeutic potential [5].

Nephrotoxicity is a well-recognised and serious adverse effect of cisplatin [3, 6, 7]. Accumulation of the drug in tubular cells results in DNA damage, oxidative stress and subsequent apoptosis [8, 9]. Cisplatin additionally induces a reduction in renal blood flow, resulting in acute kidney injury (AKI) and furthering tubular damage [8]. Such nephrotoxicity can persist after treatment cessation [3, 9, 10], and this most commonly manifests as reduced glomerular filtration rate (GFR) and chronic kidney disease (CKD), and hypomagnesaemia occasionally accompanied by secondary hypocalcaemia [11, 12]. Most cases of mild hypomagnesaemia are asymptomatic, but ventricular arrhythmia and neuromuscular irritability can occur at levels < 0.5 mmol/L [13].

A recent Cochrane review of renal sequalae after childhood cancer treatment reported a broad prevalence, ranging between 0 and 84% [14]. This variance was postulated to be related to differences in methodology, underlying malignancy and prescribed treatment. Although cisplatin was reported as a risk factor for nephrotoxicity in the majority of included studies, no subgroup analysis was performed in a cisplatin population. To date, there has been no systematic review examining the prevalence of, or risk factors for, cisplatin-induced nephrotoxicity in a paediatric population. A recent priority setting partnership between paediatric oncology patients, families and healthcare professionals in the UK listed understanding the long-term impact of antineoplastic therapies as one of the top ten research priorities within the field [15], making the issue of long-term nephrotoxicity especially relevant. Thus, this systematic review and meta-analysis was conducted to clarify the epidemiology of long-term cisplatin-induced nephrotoxicity and to understand the discrepancies in the current literature.

## Methods

This systematic review was conducted in accordance with PRISMA (Preferred Reporting Items for Systematic Reviews and Meta-Analysis) guidelines [16]. The review was registered with the PROSPERO International Prospective Register of Systematic Reviews (CRD42022301721). The protocol was amended prior to title and abstract screening to investigate long-term nephrotoxicity only, as it was felt that including acute nephrotoxicity would dilute the focus of the review. The search strategy was developed with input from information specialists and librarians within the university (see Supplementary Table 1). Searches of EMBASE, MEDLINE, Cochrane and CINAHL databases were completed on 6 January 2022.

### Eligibility criteria, screening and assessment

For studies to be eligible, they were required to: (i) investigate participants diagnosed with cancer aged < 18 years old at diagnosis; (ii) perform any author-defined method of evaluating nephrotoxicity or kidney function at least 3 months after cisplatin cessation; and (iii) be published in a peer reviewed journal. Studies whose population was broader remained eligible so long as analysis was performed in an appropriate subgroup. Case series, case reports, reviews, editorial letters and abstract-only publications were excluded. Non-English papers were initially included, although were later excluded due to difficulties in sourcing appropriate translations.

There is no population- or disease-specific definition as to what is considered “long-term” in children with chemotherapy-induced nephrotoxicity. Dysfunction persisting beyond 3 months is used to define chronic kidney disease by the Kidney Disease: Improving Global Outcomes (KDIGO) guidelines [17], a time point not contested in literature discussing the adaptation of the guidelines for paediatric populations [18, 19] and for drug-induced kidney injury [20]. Therefore, a threshold of 3 months was felt to be appropriate and clearly distinct from acute cisplatin-induced nephrotoxicity which occurs and resolves within a matter of days of dosing [21].

Search results were imported into the web-based tool Rayyan [22] to facilitate study screening. Duplicates were excluded first before two independent reviewers (JS and MH) completed title and abstract screening. Studies then underwent full-text screening by the same two independent reviewers (JS and MH). Disagreements as to inclusion suitability were resolved through discussion with a third independent reviewer (SM).

Methodological quality of included studies was assessed using the Newcastle–Ottawa Scale (NOS) for cohort studies and an adapted version of the NOS for cross-sectional studies [23] (see supplementary document 1) by one reviewer (JS). The adapted version of the NOS for cross-sectional studies was chosen as follows: (i) no gold standard exists for assessing quality of cross-sectional studies; (ii) it has been previously demonstrated to be comparable to other cross-sectional study specific appraisal tools in an oncological setting [24]; and (iii) to allow easier comparison of the quality of included studies. To facilitate use in the setting of treatment-induced nephrotoxicity in a paediatric oncology population, the list of confounding factors in Sect. 1b was amended to reflect appropriate confounding factors for the development of nephrotoxicity using findings from a 2019 Cochrane review on nephrotoxicity in CCS [25].

### Data extraction and analysis

Data extraction was completed by one reviewer (JS) and entered into an electronic data extraction form, the components of which can be found in Supplementary Table 2. Quantitative analysis was planned with a statistician (AJ). Prevalence data was combined in a meta-analysis when studies were sufficiently similar in methodology and definition of nephrotoxicity. Studies investigating GFR were analysed as one homogeneous outcome, whether they used measured or estimated GFR. When discussing these studies in general, we use the term “GFR”. When discussing specific studies using estimated GFR, we use the term “eGFR”, and where a study has measured GFR, we use “mGFR”. The *I*^2^ statistic was used to express heterogeneity, with 25%, 50% and 75% denoting low, medium and high heterogeneity respectively [26]. Only studies using the conventional threshold of < 90 ml/min/1.73 m^2^ were included in the meta-analysis of reduced GFR prevalence. Sensitivity analysis was conducted to evaluate how this affected analysis. Meta-analysis of pooled odds ratios was planned but not carried out due to a lack of comparable data. All quantitative analysis was performed using ReviewManager (RevMan) 5.4 software. Pooled prevalence analysis was conducted using a random effects model given heterogeneity between studies. Tables were created to display included relevant data pertaining to study population, missing data, quality rating, nephrotoxicity prevalence and statistical analysis. Narrative analysis examined heterogeneity between studies.

## Results

### Study selection and characteristics

**Fig. 1:**
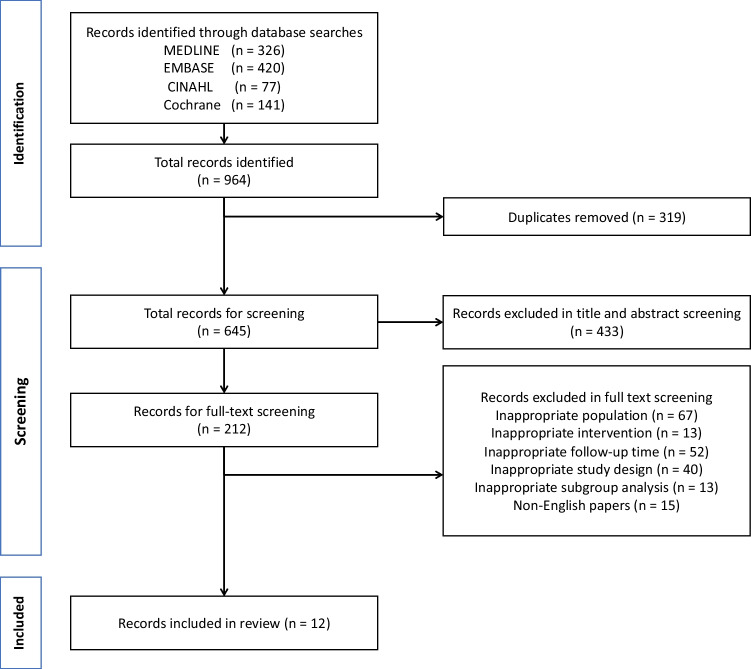
PRISMA diagram

The PRISMA flow-chart is shown in Fig. 1. Twelve studies [27–38] met inclusion criteria for the review and were published between 1991 and 2021. Studies included data from hospitals in Finland [38], the Netherlands [31, 34, 37], Poland [36], Spain [28], Turkey [27], the UK [29, 30, 33] and the USA [32, 35], although no one study contained data from more than one country. Five were cross-sectional studies [27, 31, 34, 36, 38], and seven were cohort studies [28–30, 32, 33, 35, 37]. Four studies [27, 29, 30, 32] included only participants who received cisplatin, whilst seven studies [31, 33–38] included participants treated with other anti-neoplastic regimens also, and one study [28] included healthy non-oncology controls. A total of 6512 participants were included as part of these studies, 729 of whom received cisplatin. Median age at diagnosis in the included studies ranged from 0.7 to 8.0 years. One study [32] did not state age at diagnosis, but all participants were aged < 18 years old at time of inclusion in the study. Median follow-up time ranged from 0.8 to 23.2 years, with one study [28] describing follow-up time as a mean average of 2.3 years only. The median cumulative cisplatin dose ranged from 320 to 1050 mg/m^2^.

Most studies investigated participants with a variety of underlying oncological diagnoses, aside from Pietilä et al. [38] and Canpolat et al. [32] who exclusively studied survivors of brain tumours and participants with relapsed osteosarcoma, respectively. Several large, long-term follow-up (LTFU) clinic studies [31, 34, 35, 37] did not give a breakdown of underlying diagnosis for their subgroups treated with cisplatin. Canpolat et al. [32] included some participants who were less than 3 months from completing cisplatin therapy, but the authors detailed raw data for participants, allowing the reviewers to ascertain prevalence of long-term nephrotoxicity according to the review’s definitions. Skinner et al. [33] divided participants treated with cisplatin into those who had received cisplatin and carboplatin together, those who received cisplatin but not carboplatin and those who received carboplatin and not cisplatin. Data was described in terms of these sub-groups, with no grouped data available for the overall population to receive cisplatin. Due to conflicting information published within the paper, reviewers contacted the authors of Arga et al. [27] who subsequently confirmed that participants in their study were < 18 years old at diagnosis. Methodological quality is described in Table 1.

**Table 1:**
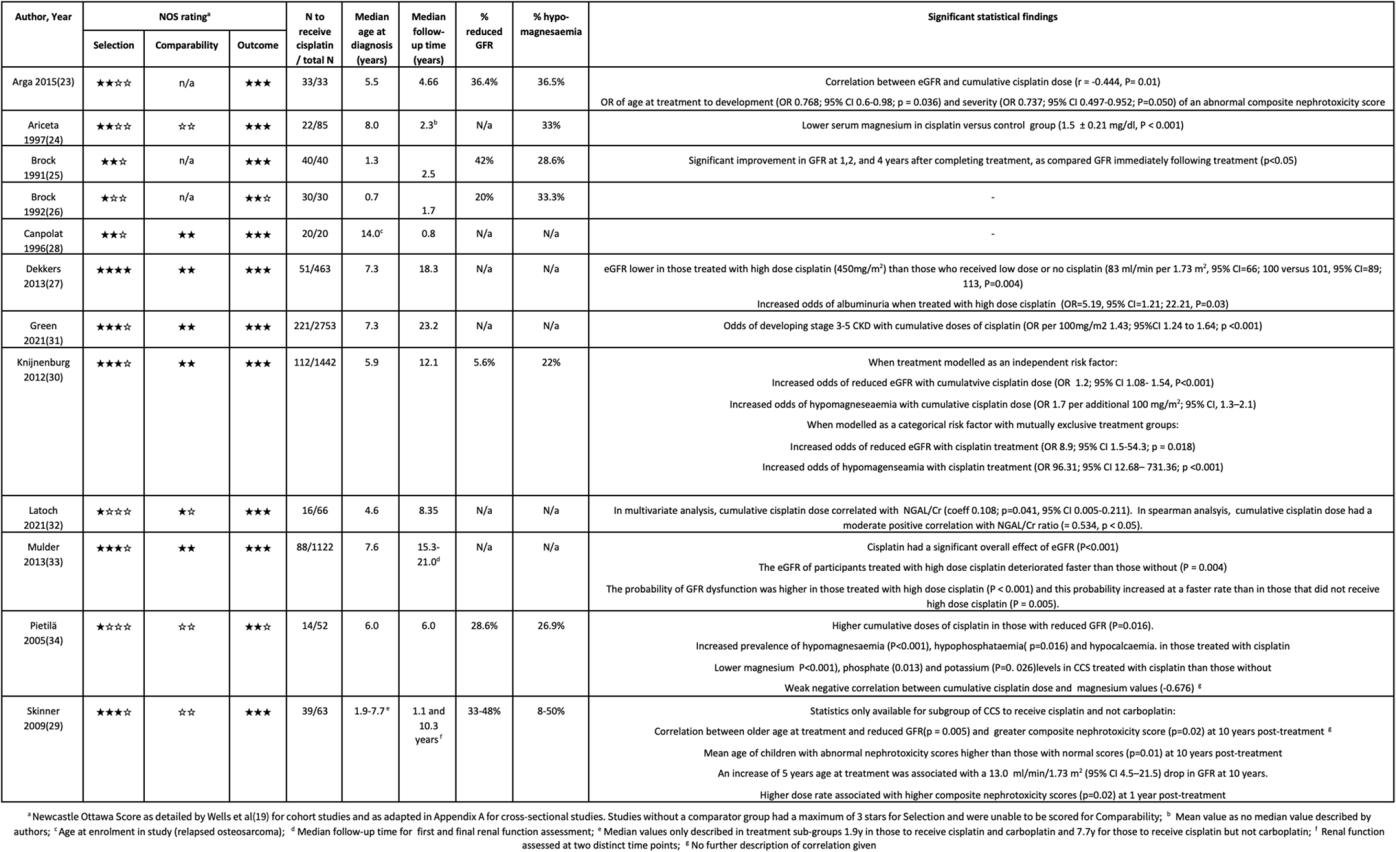
Summary of included studies

Consideration should be made for overlap in participants between included studies. The authors of two papers by Brock et al. [29, 30], both from the same team at the Hospital for Sick Children in the UK, were contacted and subsequently confirmed that there was participant overlap between the studies. The authors of the further two studies [34, 37] from the same LTFU clinic at Emma Children’s Hospital/Academic Medical Center in the Netherlands also confirmed overlap in participants in their work. The smaller, less representative of the overlapping studies was omitted from any subsequent meta-analysis.

Four studies evaluated long-term renal outcomes after cisplatin treatment in childhood, but were not able to be included in the review due to incomplete analysis within a cisplatin subgroup [39, 40], undefined length of time since treatment cessation [41] and undefined age at oncological diagnosis [42].

### Study outcomes

#### Reduced GFR

Eleven studies investigated a reduction in measured GFR (mGFR) or estimated GFR (eGFR) as a measure of nephrotoxicity. A wide variety of methods to measure and thresholds to define nephrotoxicity were used. Four studies [29, 30, 33, 38] used plasma clearance of creatinine–ethylenediaminetetraacetic acid (Cr-EDTA) to measure GFR, six studies [27, 31, 34–37] estimated GFR using serum creatinine, and one study [32] did not state their methodology. Of those studies estimating GFR using serum creatinine measurements, two used different variations of the Schwartz formula [27, 36], one used the abbreviated Modification of Diet in Renal Disease equation [31], one study used a modified version of the Schwartz formula for participants under 18 years old and the Chronic Kidney Disease Epidemiology Collaboration formula (CKDEC) for adult participants [34], and two used the CKDEC for all participants [35, 37]. In regard to thresholds, one study [32] used 60 mL/min/m^2^, two studies [29, 30] used 80 mL/min/1.73 m^2^, one study [38] used 87 mL/min/1.73 m^2^, and seven studies [27, 31, 33–37] used 90 mL/min/1.73 m^2^ as thresholds below which they defined nephrotoxicity.

Six studies [27, 29, 30, 33, 34, 38] described the prevalence of reduced GFR in participants treated with cisplatin and one further study [32] described raw data, allowing the review’s authors to calculate prevalence of reduced GFR in the long-term. Two studies [31, 37] did not provide prevalence data but did analyse cisplatin’s relationship to eGFR, and two studies [35, 36] did not provide prevalence data or further analysis of eGFR within a cisplatin population diagnosed under 18 years of age.

Prevalence of reduced GFR (shown in Supplementary Table 3) ranged from 5.6% to 48.1%. Two studies [32, 34] described nephrotoxicity notably more infrequent than other studies. Canpolat et al. [32] used a lower GFR threshold of 60 ml/min/m^2^ to define nephrotoxicity and had the shortest follow-up time of 0.8 years, which may explain why only 1 of the 17 participants had a reduced GFR. Knijnenburg et al. [34] described only 6 of 108 participants having a reduced eGFR. It is worth noting that participants in this study had the lowest median cumulative dose of cisplatin. Due to incomplete detailing of concurrent nephrotoxic therapies between papers, it was not possible to meaningfully interpret the influence of ifosfamide, high dose methotrexate and other concomitant nephrotoxic therapies on rates of reduced GFR.

**Fig. 2:**
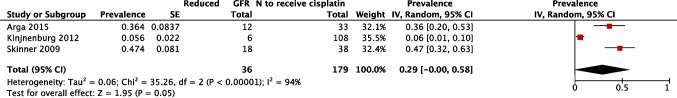
Pooled reduced GFR prevalence analysis

Three of the studies [27, 33, 34] that describe prevalence used the now conventional threshold [17] of < 90 ml/min/1.73 m^2^ to define glomerular dysfunction. Data from these three studies was used in a meta-analysis displayed in Fig. 2. Skinner et al. [33] described prevalence at 1 and 10 years. To avoid inappropriate duplication of results and given this review’s interest in nephrotoxicity in survivorship, only the 10-year values have been entered into the meta-analysis. This study also described separate prevalence for participants treated with both cisplatin and carboplatin, and those treated with cisplatin but not carboplatin. Prevalence has been combined for the meta-analysis given that other included studies did not differentiate between participants who received additional platinum-based therapies. Due consideration should be given that studies included in meta-analysis used different methods of measuring GFR, and participants differed in their age at diagnosis, cumulative cisplatin dose and follow-up time. Considering this, it is perhaps not surprising that meta-analysis showed high heterogeneity between studies with an *I*^2^ statistic of 94%. Sensitivity analysis (see Supplementary Fig. 1) was conducted to ensure that excluding studies with different thresholds for reduced GFR did not impede analysis. Including all studies with data for reduced GFR prevalence, regardless of threshold, generated a similar pooled prevalence of 0.28 (95% CI 0.12–0.44) and a similar *I*^2^ statistic of 91%.

Knijnenburg et al. [34] described a nearly ninefold increase in reduced eGFR in participants treated with cisplatin as the only nephrotoxic therapy as compared to participants treated with no nephrotoxic therapies (OR 8.9; 95% CI 1.5–54.3; *p* = 0.018). They found that cumulative cisplatin dose was linked to increased odds of having a long-term reduction in eGFR (OR per 100 mg/m^2^: 1.29; 95% CI 1.08–1.54; *p* = 0.005). Arga et al. [27] found that in 33 participants, there was a significant negative correlation between cumulative cisplatin dose and eGFR (*r* = – 0.444, *P* = 0.01). Pietilä et al. [38] found that participants with renal dysfunction had been treated with significantly higher cumulative doses of cisplatin than those without (*p* = 0.016). In a large survivorship study in the Netherlands, Dekkers et al. [31] found that eGFR was significantly lower in participants treated with high-dose (> 450 mg/m^2^) cisplatin than those treated with other regimens including low-dose cisplatin (83 ml/min/1.73 m^2^, 95% CI 66–100 versus 101 ml/min/1.73 m^2^, 95% CI 89–113; *p* = 0.004). Mulder et al. [37], publishing from the same LTFU clinic as Knijnenburg et al. [34], yielded similar findings. In a multivariable linear random effects model, they found that cisplatin had a significant overall effect on eGFR (*p* < 0.001), especially at high doses (> 500 mg/m^2^). They found the eGFR of participants treated with high-dose cisplatin deteriorated at a faster rate during the initial 25 years after diagnosis than those treated with lower doses or other regimens (*p* = 0.004). In multivariate logistic regression modelling, they found that high-dose cisplatin was associated with an increased probability of participants having a reduced eGFR (*p* < 0.001), and this probability increased at a faster rate in those treated with high-dose cisplatin compared to low-dose or other regimens during 35 years following diagnosis (*p* = 0.005). However in contrast, one study by Brock et al. [29] did not find a correlation between cumulative dose and change in mGFR. It is worth noting that this study was smaller (*n* = 40) and had a far shorter follow-up period (2.5 years).

Studies also analysed the influence of other factors on cisplatin-induced nephrotoxicity. Skinner et al. [33] found that in 27 participants treated with cisplatin but not carboplatin, there was a negative correlation between age at treatment and mGFR at 10 years post-treatment (*p* = 0.005), with an increase of 5 years in age at time of treatment being associated with a fall in mGFR of 13.0 ml/min/1.73 m^2^ (95% CI 4.5–21.5). They did not perform such analysis for the combined cisplatin and carboplatin treatment group due to small population size. None of the other included studies described age at diagnosis as being related to GFR. Two studies [29, 30], both conducted by Brock et al., showed significant improvement in mGFR at follow-up compared to that at the end of treatment. Skinner et al. [33] also found that there was an increase in mGFR over the study, although this was not statistically significant and the authors reported substantial interindividual variation. Comparing the influence of other nephrotoxic chemotherapies, Arga et al. [27] did not find a significant difference in mean average eGFR between cisplatin populations that had and had not received concurrent ifosfamide.

Ariceta et al. [28] measured serum creatinine but did not describe GFR. Six out of 18 (33.3%) participants in their study were classed as having elevated serum creatinine, although the authors did not define what threshold they used to define this.

#### Chronic kidney disease (CKD) as a diagnosis

Green et al. [35] were the only study to define nephrotoxicity in terms of a diagnosis of chronic kidney disease (CKD). The study evaluated eGFR with serum creatinine and evaluated proteinuria via morning urine dipstick and then used this data to diagnose CKD as per KDIGO criteria [17]. They did not describe CKD prevalence within a subgroup treated with cisplatin. They ran 4 different elastic net multivariate models for different doses of kidney radiation (V5-20). All models found a 1.4-fold increase in odds of developing stage 3–5 CKD per 100 mg/m^2^ of cumulative cisplatin administered (for V5 and V10: OR 1.4, 95% CI 1.25–1.65, *p* < 0.001; for V15 and V20: OR 1.43, 95% CI 1.24–1.64; *p* < 0.001).

#### Hypomagnesaemia

Eight studies [27–30, 32–34, 38] investigated hypomagnesaemia, and all but Canpolat et al. [32] described long-term prevalence in a population treated with cisplatin. All examined serum magnesium levels except for Ariceta et al. [28] and Pietilä et al. [38] who measured plasma magnesium. Different thresholds were used to define hypomagnesaemia apart from two studies by the same author [29, 30] which used the same threshold. A further two studies [32, 38] did not state the threshold used.

Prevalence of hypomagnesaemia (shown in Supplementary Table 4) ranged from 8.0 to 71.4%. The two groups [33, 38] describing the upper and lower limits of prevalence had the smallest sample sizes. Skinner et al. [33] described notably lower prevalence in their combined cisplatin and carboplatin subgroup at 1 and 10 years after treatment completion, despite their cisplatin only subgroup having similar rates to the other included studies. Participants in the combined treatment group were younger than the cisplatin only group and had a different proportion of underlying oncological diagnoses. Both studies from Brock et al. [29, 30] also included participants relatively young at diagnosis but described higher prevalence rates more in line with other studies. It is worth noting that both studies from Brock et al., along with Knijnenburg et al. [34], had a substantial proportion of participants lacking magnesium data, and their results may subsequently be subject to sampling bias. Most studies reported similar cumulative doses of cisplatin apart from Knijnenburg et al. [34] which was notably lower than the rest at 320 mg/m^2^. Due to incomplete reporting of underlying diagnoses across studies, it is not possible to interpret the role of underlying diagnosis on hypomagnesaemia. Only one small study [29] examined that relationship between mGFR and hypomagnesaemia and found no correlation between the two.

**Fig. 3:**
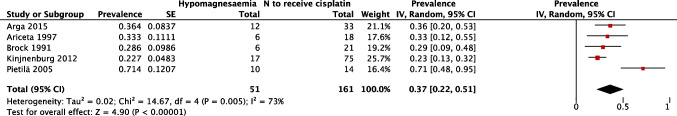
Pooled hypomagnesaemia prevalence analysis

Meta-analysis of hypomagnesaemia prevalence is shown in Fig. 3. Given the confirmed overlap in participants between the two studies by Brock et al. [29, 30], their 1992 study was omitted from the meta-analysis to avoid inappropriate duplication of data. The 1992 paper was chosen for omission as it included fewer participants and studied a non-representative population. Additionally, due to imprecise sample size for participants with magnesium data, results from Skinner et al. [33] could not be included in the meta-analysis. Due consideration should be given to this latter omission, and the different thresholds and demographic data of the included studies. Medium heterogeneity was present, with an *I*^2^ statistic of 73%.

Pietilä et al. [38] found that magnesium levels were significantly lower in participants who were treated with cisplatin than those without (0.64 versus 0.85 mmol/L, *p* < 0.001). They also found a higher prevalence of hypomagnesaemia in participants treated with cisplatin than without (*p* < 0.001) and a weak negative correlation between cumulative cisplatin dose and magnesium values, although no *p* value was given (*r* = – 0.676). Ariceta et al. [28] found similar results with significantly lower magnesium levels in participants treated with cisplatin than their healthy non-oncology control population (1.5 ± 0.21 mg/dl versus 1.70 ± 0.15 mg/dl, *p* < 0.001). However, they did not establish any correlation between magnesium level and cumulative dose, and they did not find a correlation with time elapsed since treatment either.

In multivariate analysis, Knijnenburg et al. [34] found that treatment with cisplatin was associated with a 96-fold increase in odds for developing hypomagnesaemia (OR 96.31, 95% CI 12.68–731.36, *p* < 0.001) compared to those treated with no nephrotoxic therapies. Furthermore, they found that this risk was associated with increasing cumulative dose (OR 1.7 per 100 mg/m^2^; 95% CI 1.3–2.1, *p* < 0.001). Querying the effect of additional nephrotoxic therapies, Arga et al. [27] did not find any significant difference between cisplatin populations who had and had not received ifosfamide.

#### Other electrolyte disturbance

Five studies [27, 29, 30, 33, 38] reported on hypocalcaemia, four studies [27–29, 38] on hypokalaemia and four studies [27, 32, 34, 38] on hypophosphataemia. The results are shown in Supplementary Tables 5, 6 and 7. Several studies performed statistical analysis regarding the relationship between these outcome variables and cisplatin treatment. Pietilä et al. [38] found no difference in plasma calcium values between participants treated with or without cisplatin. All participants studied by Arga et al. [27] received cisplatin, but authors found no difference in serum calcium levels between participants treated with cisplatin with or without ifosfamide. They also did not find any difference in serum potassium between these groups. Pietilä et al. [38] did find serum potassium to be marginally but significantly lower in participants who received cisplatin than those without, although the median value for both remained within normal limits (3.7 mmol/L versus 3.8 mmol/L, *p* = 0.026). They did not find participants treated with cisplatin to have significantly higher rates of hypokalaemia (*p* = 0.269).

When looking at phosphate, Arga et al. [27] found that hypophosphataemia was associated with combined treatment with ifosfamide compared to cisplatin alone, with all 4 participants who experienced hypophosphataemia receiving combination treatment. Pietilä et al. [38] found that participants treated with cisplatin had significantly lower plasma phosphate levels (1.09 mmol/L versus 1.32 mmol/L, *p* = 0.013) and significantly higher rates of hypophosphatemia (*p* = 0.016). None of the participants in Pietilä et al. [38] are described as having received concurrent ifosfamide treatment. In multivariate analysis from a large survivorship clinic study in the Netherlands, Knijnenburg et al. [34] did not find cumulative dosing of cisplatin to be a risk factor for hypophosphataemia (OR per 100 mg/m^2^: 1.00; 95% CI 0.77–1.30, *p* = 0.99).

#### Proteinuria

Five studies [31, 34–36, 38] investigated proteinuria utilising a variety of methods. Two studies [34, 35] used urine dipstick [34, 35], one study used urinary albumin concentration [36], one study used urinary albumin:creatine ratio [31], and one study looked at urinary protein concentration and performed a 24-h urine collection if > 0.1 g/L [38]. All studies used different thresholds for defining proteinuria. Three of the five studies [31, 34, 38] described prevalence in a population appropriate for inclusion in this review, ranging from 7.1 to 17.9% (see Supplementary Table 8).

Included studies described conflicting findings regarding the relationship between cisplatin and proteinuria. Pietilä et al. [38] did not find a significant difference in proteinuria between the 14 participants treated with cisplatin and 38 participants treated with other regimens (*p* = 1.000). In a large LTFU study from the USA, Green et al. [35] also did not find cisplatin to be a risk factor for proteinuria in univariate analysis, either as a categorical variable or in terms of cumulative dose. In a further LTFU in the Netherlands, Knijnenburg et al. [34] also did not find an association between cisplatin and albuminuria when looking at participants treated with cisplatin as the only nephrotoxic therapy compared to participants treated with no nephrotoxic antineoplastic therapies at all (OR 2.20; 95% CI 0.94–5.14; *p* = 0.07). They did not find cumulative cisplatin dose to be a treatment-related risk factor for albuminuria.

Contrastingly, in a study from another survivorship clinic in the Netherlands, Dekkers et al. [31] did find that treatment with high-dose cisplatin (> 450 mg/m^2^) was significantly and independently associated with albuminuria (OR: 5.19, 95% CI 1.21–22.21, *p* = 0.03), whilst low-dose cisplatin was not. It is worth noting that no other included study differentiated between high- and low-dose cisplatin as categorical variables when evaluating proteinuria.

#### Composite scores

Two studies [27, 33] described nephrotoxicity as a composite score. Both used the same score to grade participants according to the severity of GFR and magnesium level derangement, with Arga et al. [27] directly referencing that first used by Skinner et al. [33]. The scoring criteria are displayed in Supplementary Table 9. The scores for GFR and magnesium were combined to give an overall nephrotoxicity score with 1 denoting mild, 2–3 moderate and ≥ 4 severe nephrotoxicity. Note should be made that the studies still differed in their methods for measuring GFR and magnesium as detailed elsewhere in this review.

As with other nephrotoxicity measures, Skinner et al. [33] described rates of nephrotoxicity in treatment and follow-up subgroups only. Prevalence of nephrotoxicity in the two studies, found in Supplementary Table 10, ranged from 6.1 to 31.8% depending on the grade of nephrotoxicity, follow-up time and treatment subgroup.

When conducting analysis regarding risk factors for composite nephrotoxicity, both studies separated the scores into dichotomous categories of “normal” for scores 0–1 and abnormal for scores of ≥ 2. This meant that participants who scored 1 for either mildly reduced GFR or mild hypomagnesemia were still classed as “normal” in their categorical analyses. Arga et al. [27] did not find a significant difference in composite nephrotoxicity between participants treated with cisplatin alone and those treated with concurrent ifosfamide or radiotherapy. In multivariate analysis, they did not find cisplatin dose or time elapsed since treatment to be risk factors for the development of, or severity of, nephrotoxicity. However, they did find age at treatment related to both development (OR 0.768, 95% CI 0.6–0.98, *p* = 0.036) and severity (OR 0.737, 95% CI 0.497–0.952, *p* = 0.050) of nephrotoxicity, with younger age at treatment being associated with poor outcomes.

In direct conflict, Skinner et al. [33] found that older age at treatment correlated with greater overall nephrotoxicity (*p* = 0.02) at 10 years after treatment in the subgroup who received cisplatin and not carboplatin, with the mean age of children with abnormal scores being significantly higher than those with normal scores (*p* = 0.01). They did not state whether this was the case at 1-year post-treatment, and they did not carry out such analysis in the subgroup who received cisplatin and carboplatin as the sample size was too small. The authors of Arga et al. [27] postulated whether the difference between the study’s findings was related to a third of their cohort receiving concurrent treatment with ifosfamide, whilst no participant in Skinner et al.’s study did. Skinner et al. [33] did not find association between overall dose and composite nephrotoxicity, but did find and a relationship between dose rate and higher composite nephrotoxicity scores (*p* = 0.02) at 1 year post-treatment.

#### Hypertension

Eight studies [27, 29, 31, 33–36, 38] reported on hypertension as a measure of nephrotoxicity. Studies varied as to their methodology and thresholds for defining hypertension (see Supplementary Table 11), with many studies not stating one or both pieces of information. Prevalence in participants who received cisplatin was not reported at all in two studies [35, 36]. A further two studies [29, 33] stated how many participants had hypertension but did not state how many participants had their blood pressure measured. When reported, prevalence ranged from 0.0 to 42.9%. Pietilä et al. [38], who reported the highest prevalence of 42.9%, studied only brain tumour survivors and scored poorly on the NOS.

Two studies [34, 38] performed statistical analysis examining cisplatin’s relationship to hypertension. In multivariate analysis, Knijnenburg et al. [34] did not find cisplatin to be a risk factor for the development of hypertension when examined according to cumulative dose (OR per 100 mg/m^2^ 1.13, 95% CI 0.98–1.28, *p* = 0.09) or as a categorical variable in mutually exclusive antineoplastic treatment groups (OR 0.83, 95%CI 0.30–2.29, *p* = 0.72). Contrastingly, in a smaller study exclusively of those treated for brain tumours, Pietilä et al. [38] found that hypertension was more common in participants treated with cisplatin than those without (*p* = 0.003) especially if participants received concurrent cranial irradiation (*p* = 0.002).

#### Other author defined nephrotoxicity

Four studies [27, 31, 36, 38] reported on other outcome variables described as measures of nephrotoxicity, including serum cystatin C, urinary alpha-1-microglobulin, urinary beta-2-microglobulin:creatinine ratio, renal threshold for phosphate and new renal biomarkers neutrophil gelatinase-associated lipocalin (NGAL) and kidney injury molecule 1 (KIM-1). The results are shown in Supplementary Table 12. Latoch et al. [36] described a significant positive correlation between NGAL:creatinine ratio (NGAL/Cr) and cumulative cisplatin dose.

## Discussion

To our best knowledge, this is the first meta-analysis of long-term cisplatin nephrotoxicity after treatment in childhood. Prevalence of reduced GFR ranged between 5.9 and 48.1% and the meta-analysis of studies using the modern consensus threshold of 90 ml/min/1.73 m^2^ gave a pooled prevalence of 29% (95% CI 0.0–58%). Prevalence of hypomagnesaemia ranged between 8.0 and 71.4% and the meta-analysis gave a pooled prevalence of 37% (95% CI 22–51%). However, due consideration should be given to the substantial heterogeneity between studies reflected in the *I*^2^ statistics of 94% and 73% for reduced GFR and hypomagnesaemia respectively. This heterogeneity is likely related to small population sizes and variation in participant demographics, follow-up time, methodology, and cisplatin dosing. Some of this heterogeneity may be inevitable and reflects the different dosing protocols for, and different age groups susceptible to, the various cancers for which cisplatin is a common treatment. The relationship between reduced GFR, hypomagnesaemia and other manifestations of nephrotoxicity remains uncharacterised.

When evaluating risk factors for cisplatin-nephrotoxicity, the most prominent influence was that of cumulative cisplatin dose. Although not all small studies reported a relationship [33], all four large, high-quality, LTFU studies [31, 34, 35, 37] described higher cumulative cisplatin dose as conferring increased risk of developing reduced GFR, hypomagnesaemia and/ or stage 3–5 CKD. There was conflicting evidence as to whether this extended to proteinuria, and additional work differentiating categorically between populations receiving high- and low-dose cisplatin is needed to understand this.

Two other risk factors were identified in the included studies evaluating composite nephrotoxicity scores: age at treatment and dose rate. Dose rate was only evaluated by one small study [33], but faster rates were found to be associated with increased nephrotoxicity in some treatment subgroups. Two studies [27, 33], similar in quality, size and follow-up produced conflicting data as to whether younger or older age at treatment conferred an increased risk of nephrotoxicity. Further work exploring both these and other factors not investigated in the included studies, such as underlying malignancy and concurrent nephrotoxic therapies, is needed.

Kidney function gradually declines during adulthood in healthy populations [43]. The review yielded some low-quality evidence that GFR may actually improve immediately following treatment cessation [29, 33], similar to that reported in adults treated with the drug [44]. However, any such recovery may be short-lived. One high-quality LTFU study [37] evaluating GFR time-trends up to 35 years after diagnosis found that CCS who received high-dose cisplatin suffered faster deterioration in kidney function than their CCS counterparts who received other regimens. It is unknown whether hypomagnesaemia follows a similar pattern.

Despite hypertension being a recognised complication of renal compromise, it was poorly reported amongst included studies. One poor quality study of brain tumour survivors [38] did suggest an association between hypertension and cisplatin whilst a large high-quality LTFU study did not [34]. Further investigation of the issue is needed, with appreciation that vascular sequalae of childhood cancer can also contribute to hypertension in the survivorship period [12].

Other incongruencies were noted in this review regarding hypophosphataemia. Hypophosphataemia is classically associated with ifosfamide treatment [12], and it is thus unsurprising that hypophosphataemia was associated with combined cisplatin and ifosfamide treatment [27]. One study [38] investigating brain tumour survivors identified a significant association between hypophosphataemia and cisplatin in a population that did not receive concurrent ifosfamide. It is unknown whether this relates to underlying diagnosis, poor methodological quality or a potential absence of ifosfamide treatment allowing subtle changes in phosphate relating to cisplatin to be elucidated.

### Limitations

This review includes studies from 8 countries across Europe, North America and Asia. No studies produced data from developing healthcare systems, and this limits generalisability in these settings. Appropriate translations were not able to be sourced for non-English papers, and this further limits the scope and generalisability of the review.

Similar to a broader Cochrane review on renal outcomes after childhood cancer [25], this review was impeded by marked heterogeneity in included studies’ methodology. Some issues such as varying threshold definitions for reduced GFR are unlikely to recur in future work given modern consensus guidelines [17]. Consensus guidelines for methodology regarding measurement of GFR and other forms of nephrotoxicity in CCS LTFU could theoretically resolve the issue of heterogeneity, although real-world application of this may be constrained by established local laboratory practice. Further heterogeneity in statistical analysis methods prevented meta-analysis of pooled odds ratios, restricting risk factor analysis to narrative only.

### Recommendations and future directions

Current LTFU protocols recommend non-stratified kidney function surveillance for all CCS treated with cisplatin. Given the findings of this review, it may be prudent to recommend more frequent monitoring in CCS treated with cumulative cisplatin doses ≥ 450 mg/m^2^. Future work would be beneficial in clarifying the role of demographic-related risk factors such as age at treatment, as well as identifying other factors associated with susceptibility to nephrotoxicity to allow further stratification. Genetic variants confer increased vulnerability to cisplatin ototoxicity in children treated with the drug [45], and similar variants for cisplatin nephrotoxicity have been studied in adults [46, 47]. It is likely, therefore, that there may also be genetic susceptibility to cisplatin nephrotoxicity in the paediatric population, and these CCS may warrant closer surveillance should such genetic variants be identified.

Multiple LTFU studies of nephrotoxicity in CCS did not specifically describe nephrotoxicity prevalence in a cisplatin-exposed subgroup and instead only described prevalence in an overall CCS population. Going forward, similar studies should consider reporting prevalence in treatment-specific subgroups. Whilst not impervious to potentially confounding concurrent nephrotoxic therapies, treatment-specific prevalence rates would allow a better understanding of risk when making treatment decisions and counselling families*.*

The papers included in this review did not establish a relationship between the glomerular and tubular manifestations of long-term nephrotoxicity, or give any insight into the sequence of their development. Future work elucidating these issues would improve understanding of the aetiological mechanism of the disease process and thus possibly allow earlier intervention. Novel biomarkers may also play a role in this regard. NGAL was identified as an emerging biomarker in one of the studies included in this review. Beyond contributing to an aetiological understanding, future work evaluating its role and temporality in cisplatin nephrotoxicity may also contribute to the development of targeted preventative therapies.

## Conclusion

In conclusion, this meta-analysis was impeded by heterogeneity but suggests that 29% of children treated with cisplatin will develop a reduction in GFR and 37% will develop hypomagnesaemia—the most common manifestations of long-term cisplatin nephrotoxicity. This nephrotoxicity relates to cumulative cisplatin dose, with increased doses conferring increased risk. Further work is needed to understand the risk posed by dose rate and age at treatment, as well as other potential manifestations of cisplatin nephrotoxicity such as proteinuria and hypertension. Future studies should also consider investigating the relationship between different manifestations of toxicity, in addition to genetic vulnerability and novel biomarkers for long-term cisplatin nephrotoxicity.

### Supplementary Information

Below is the link to the electronic supplementary material.

Supplementary file1 (DOCX 460 KB)

## Declarations

### Disclaimer

The views expressed in this publication are those of the author(s) and not necessarily those of the NIHR, NHS or the UK Department of Health and Social Care.
