# Supplementary material for: Long-term cisplatin nephrotoxicity after childhood cancer: a systematic review and meta-analysis
Source: Pediatr Nephrol. 2023 Sep 20;39(3):699–710. doi: 10.1007/s00467-023-06149-9 (PMC10817831; doi:10.1007/s00467-023-06149-9)
Supplement: Supplementary file 1 — Supplementary file1 (DOCX 460 KB) [file 467_2023_6149_MOESM1_ESM.docx]

# Supplementary Table 1

| **Database** | **Search Terms** |
| --- | --- |
| MEDLINE filtering for human studies only | (Cisplatin* OR "Platinum Diamminodichloride" OR "cis Platinum" OR Dichlorodiammineplatinum OR "cis Diamminedichloroplatinum" OR "cis Dichlorodiammineplatinum(II)" OR Platinol OR Platidiam OR Platino OR NSC119875 OR "NSC 119875" OR Biocisplatinum OR CDDP OR CACP OR abiplatin OR neoplatin OR cisDDP OR "antineoplastic agent*" OR "cancer treatment*").ti,ab OR *CISPLATIN/ OR *"ANTINEOPLASTIC AGENTS"/  AND  (Nephrotoxicity OR nephrotoxic OR "renal toxicity" OR "renal injury" OR "acute renal injury" OR "kidney injury" OR "acute kidney injury" OR "acute kidney disease" OR AKI OR "kidney failure" OR "acute renal failure" OR ARF OR "Renal insufficiency" OR "kidney insufficiency" OR CKD OR "renal disease" OR "kidney disease" OR "glomerular filtration rate" OR hypomagnesemia OR hypomagneseamia OR hypophosphatemia OR hypophosphataemia OR "Fanconi syndrome" OR hypocalcemia OR hypocalcaemia OR hypokalemia OR hypokalaemia OR proteinuria OR "electrolyte disturbance" OR "electrolyte wasting").ti,ab OR *"ACUTE KIDNEY INJURY"/ OR *"RENAL INSUFFICIENCY"/ OR *"KIDNEY FAILURE, CHRONIC"/  AND  (pediatric OR paediatric OR child* OR infan* OR newborn* OR new-born OR neonate* OR neonatal OR adolescent* OR teenage* OR juvenile).ti,ab |
| EMBASE with filter for human studies only | (Cisplatin* OR "Platinum Diamminodichloride" OR "cis Platinum" OR Dichlorodiammineplatinum OR "cis Diamminedichloroplatinum" OR "cis Dichlorodiammineplatinum(II)" OR Platinol OR Platidiam OR Platino OR NSC119875 OR "NSC 119875" OR Biocisplatinum OR CDDP OR CACP OR abiplatin OR neoplatin OR cisDDP OR "antineoplastic agent*" OR "cancer treatment*").ti,ab OR *CISPLATIN/ OR *"ANTINEOPLASTIC AGENTS"/  AND  (Nephrotoxicity OR nephrotoxic OR "renal toxicity" OR "renal injury" OR "acute renal injury" OR "kidney injury" OR "acute kidney injury" OR "acute kidney disease" OR AKI OR "kidney failure" OR "acute renal failure" OR ARF OR "Renal insufficiency" OR "kidney insufficiency" OR CKD OR "renal disease" OR "kidney disease" OR "glomerular filtration rate" OR hypomagnesemia OR hypomagneseamia OR hypophosphatemia OR hypophosphataemia OR "Fanconi syndrome" OR hypocalcemia OR hypocalcaemia OR hypokalemia OR hypokalaemia OR proteinuria OR "electrolyte disturbance" OR "electrolyte wasting").ti,ab OR *"ACUTE KIDNEY INJURY"/ OR *"RENAL INSUFFICIENCY"/ OR *"KIDNEY FAILURE, CHRONIC"/  AND  (pediatric OR paediatric OR child* OR infan* OR newborn* OR new-born OR neonate* OR neonatal OR adolescent* OR teenage* OR juvenile).ti,ab |
| CINAHL | (Cisplatin* OR "Platinum Diamminodichloride" OR "cis Platinum" OR Dichlorodiammineplatinum OR "cis Diamminedichloroplatinum" OR "cis Dichlorodiammineplatinum(II)" OR Platinol OR Platidiam OR Platino OR NSC119875 OR "NSC 119875" OR Biocisplatinum OR CDDP OR CACP OR abiplatin OR neoplatin OR cisDDP OR "antineoplastic agent*" OR "cancer treatment*").ti,ab or *CISPLATIN/ OR *"ANTINEOPLASTIC AGENTS"/  AND  (Nephrotoxicity OR nephrotoxic OR "renal toxicity" OR "renal injury" OR "acute renal injury" OR "kidney injury" OR "acute kidney injury" OR "acute kidney disease" OR AKI OR "kidney failure" OR "acute renal failure" OR ARF OR "Renal insufficiency" OR "kidney insufficiency" OR CKD OR "renal disease" OR "kidney disease" OR "glomerular filtration rate" OR hypomagnesemia OR hypomagneseamia OR hypophosphatemia OR hypophosphataemia OR "Fanconi syndrome" OR hypocalcemia OR hypocalcaemia OR hypokalemia OR hypokalaemia OR proteinuria OR "electrolyte disturbance" OR "electrolyte wasting").ti,ab OR *"ACUTE KIDNEY INJURY"/ OR *"RENAL INSUFFICIENCY"/ OR *"KIDNEY FAILURE, CHRONIC"/  AND  (pediatric OR paediatric OR child* OR infan* OR newborn* OR new-born OR neonate* OR neonatal OR adolescent* OR teenage* OR juvenile).ti,ab |
| Cochrane | Cisplatin* OR "Platinum Diamminodichloride" OR "cis Platinum" OR Dichlorodiammineplatinum OR "cis Diamminedichloroplatinum" OR "cis Dichlorodiammineplatinum(II)" OR Platinol OR Platidiam OR Platino OR NSC119875 OR "NSC 119875" OR Biocisplatinum OR CDDP OR CACP OR abiplatin OR neoplatin OR cisDDP OR "antineoplastic agent*" OR "cancer treatment*" OR MeSH descriptor: [Cisplatin] this term only  AND  Nephrotoxicity OR nephrotoxic OR "renal toxicity" OR "renal injury" OR "acute renal injury" OR "kidney injury" OR "acute kidney injury" OR "acute kidney disease" OR AKI OR "kidney failure" OR "acute renal failure" OR ARF OR "Renal insufficiency" OR "kidney insufficiency" OR CKD OR "renal disease" OR "kidney disease" OR "glomerular filtration rate" OR hypomagnesemia OR hypomagneseamia OR hypophosphatemia OR hypophosphataemia OR "Fanconi syndrome" OR hypocalcemia OR hypocalcaemia OR hypokalemia OR hypokalaemia OR proteinuria OR "electrolyte disturbance" OR "electrolyte wasting" OR MeSH descriptor: [Acute Kidney Injury] this term only OR MeSH descriptor: [Renal Insufficiency] this term only  AND  pediatric OR paediatric OR child* OR infan* OR newborn* OR new-born OR neonate* OR neonatal OR adolescent* OR teenage* OR juvenile |

*Supplementary table 1: Search terms for each database searched*

# Supplementary Table 2

| **Data items for extraction** | |
| --- | --- |
| **Study Characteristics** | First author, Year of publication, Journal, Design, Follow-up time, Total number of participants, Number of participants who received cisplatin, Withdrawals / incomplete data, Setting, Start date, End date, Study's inclusion criteria, Study's exclusion criteria, Primary outcome, Statistical analysis performed, Study sponsorship, Recruitment procedures, Conflicts of Interest |
| **Total study population** | Age at diagnosis, Age at investigation, Gender breakdown, ethnicity breakdown, Baseline co-morbidities, Cancer diagnosis breakdown, Renal tumour proportion, Cancer characteristics, Complications breakdown, Treatment with carboplatin treatment, Treatment with ifosfamide treatment, Treatment with high-dose methotrexate, Treatment with melphalan, Treatment with cyclophosphamide, Treatment with abdominoperitoneal / total body radiation, Treatment with nephrotoxic antimicrobial treatment, Treatment with contrast media, Treatment with nephrectomy, Treatment with any nephrotoxic chemotherapy (and study definition of nephrotoxic treatment). |
| **Population to receive cisplatin** | Number of patients receiving cisplatin, Cisplatin dose (median), cisplatin dose (range), Cisplatin schedule, Nephroprotective measures, Complications breakdown, Concurrent carboplatin treatment, Concurrent ifosfamide treatment, Concurrent high-dose methotrexate, Concurrent melphalan, Concurrent cyclophosphamide, Concurrent abdominoperitoneal / total body radiation, Concurrent nephrotoxic antimicrobial treatment, Concurrent contrast media, Concurrent nephrectomy, Concurrent with any other nephrotoxic chemotherapy (and study definition of nephrotoxic treatment). |
| **Control population** | Number of controls, Age of control pop, Recruitment source, Comparability between populations (including statistical significance) |
| **Specific measures of nephrotoxicity – individual data points on the right hand column were collected for (Serum creatinine elevation; reduction in glomerular filtration rate; hypomagnesaemia, hypokalaemia, hypocalcaemia, hypophosphataemia, proteinuria, hypertension, hypertension, chronic kidney disease as a diagnosis)** | Method of measurement, Threshold used to define toxicity, When was measurement performed, Average value for nephrotoxicity measure in total population, Average value for nephrotoxicity measure in population to receive cisplatin, Average value for nephrotoxicity measure in control population, Prevalence of nephrotoxicity in total study population, Prevalence of nephrotoxicity in population to receive cisplatin, Prevalence of nephrotoxicity in control population, Statistical analysis pertaining to cisplatin’s relationship with nephrotoxicity. |
| **Other author defined methods of nephrotoxicity not defined prior to extraction** | Method of measurement, Threshold used to define toxicity, When was measurement performed, Average value for nephrotoxicity measure in total population, Average value for nephrotoxicity measure in population to receive cisplatin, Average value for nephrotoxicity measure in control population, Prevalence of nephrotoxicity in total study population, Prevalence of nephrotoxicity in population to receive cisplatin, Prevalence of nephrotoxicity in control population, Statistical analysis pertaining to cisplatin’s relationship with nephrotoxicity (including measures of significance, confidence and what factors were adjusted for in any multivariable analysis). |

*Supplementary table 2: Items for data extraction*

# Supplementary Table 3

| **Study** | **Prevalence (%)** | **N to have GFR data / N to receive cisplatin** | **Threshold** | **Method** | **Median follow-up time (years)** | **Median cumulative dose (mg/m2)** | **Median age at diagnosis** |
| --- | --- | --- | --- | --- | --- | --- | --- |
| Arga 2015(23) | 36.4 | 33/33 | <90ml/min/1.73 m^2^ | Serum creatinine & Schwartz formula | 4.7 | 500 | 5.5 |
| Brock 1991(25) | 42.5 | 40/40 | <80ml/min/1.73 m^2^ | Cr-EDTA | 2.5 | 500 | 1.3 |
| Brock 1992(26) | 20.0 | 15/30 | <80ml/min/1.73 m^2^ | Cr-EDTA | 1.7 | 400 | 0.7 |
| Canpolat 1996(28) | 5.9 | 17/20 | <60ml/min/m^2^ | Not stated | 0.8 | 1050 | Not stated |
| Skinner 2009 CPL 1y(29) | 42.8 | 21/21 | <90ml/min/1.73m^2^ | Cr-EDTA | 1.1 | 500 | 7.7 |
| Skinner 2009 CPL 10y(29) | 48.1 | 27/27 | <90ml/min/1.73m^2^ | Cr-EDTA | 10.3 | 500 | 7.7 |
| Skinner 2009 CPL + CBP 1y(29) | 33.3 | 12/12 | <90ml/min/1.73m^2^ | Cr-EDTA | 1.1 | 473 | 1.9 |
| Skinner 2009 CPL + CBP 10y(29) | 45.5 | 11/12 | <90ml/min/1.73m2 | Cr-EDTA | 10.3 | 473 | 1.9 |
| Knijnenburg 2012(30) | 5.6 | 108/112 | <90ml/min/1.73m^2^ | Serum creatinine & adapted Schwartz formula if <18 y/o at time of testing or CKDEC formula for adult survivors. | 12.1 | 320 | 5.9 |
| Pietilä 2005(34) | 28.6 | 14/14 | <87ml/min/1.73m^2^ | Cr-EDTA | 6.0 | 528 | 6.0 |
| Dekkers 2013(27) | Not stated | N/a | <90ml/min/1.73m^2^ | Serum creatinine & abbreviated Modification of Diet in Renal Disease equation | 18.3 | 450 | 7.3 |
| Green 2021(31) | Not stated | N/a | <90ml/min/1.73m^2^ | Serum creatinine & CKDEC formula | 23.2 | 398 | 7.3 |
| Latoch 2021(32) | Not stated | N/a | <90ml/min/1.73m^2^ | Serum creatinine & updated Schwartz formula | 8.35 | 400 | 4.6 |
| Mulder 2013(33) | Not stated | N/a | <90ml/min/1.73m^2^ | Serum creatinine & CKDEC formula | 15.3-21.1^a^ | 365 | 7.6 |
| ^a^ Median follow-up time for first and final renal function assessment | | | | | | | |

*Supplementary table 3: Summary table for reduced GFR*

# Supplementary Table 4

| **Study** | **Prevalence** **(%)** | **N to have magnesium data / N to receive cisplatin** | **Threshold** | **Median follow-up time (years)** | **Median cumulative dose (mg/m^2^)** | **Median age at diagnosis** |
| --- | --- | --- | --- | --- | --- | --- |
| Arga 2015(23) | 36.5 | 33/33 | < 0.7mmol/L | 4.7 | 500 | 5.5 |
| Ariceta 1997(24) | 33.3 | 18/22 | < 1.4 mg/dl | 2.3 | 570 | 8.0 |
| Brock 1991(25) | 28.6 | 21/40 | < 0.75mmol/L in infants; results >2 SD below mean average beyond this age group | 2.5 | 500 | 1.3 |
| Brock 1992(26) | 33.3 | 15/30 | 0.75mmol/L in infants; results >2 SD below mean average beyond this age group | 1.7 | 400 | 0.7 |
| Skinner 2009 CPL 1y(29) | 50.0 | Imprecise values supplied | <0.75mmol/L if < 2 years old <0.7mmol/L if ≥ 2 years old | 1.1 | 500 | 7.7 |
| Skinner 2009 CPL 10y(29) | 32.0 | Imprecise values supplied | <0.75mmol/L if < 2 years old <0.7mmol/L if ≥ 2 years old | 10.3 | 500 | 7.7 |
| Skinner 2009 CPL + CBP 1y(29) | 8.0 | Imprecise values supplied | <0.75mmol/L if < 2 years old <0.7mmol/L if ≥ 2 years old | 1.1 | 473 | 1.9 |
| Skinner 2009 CPL + CBP 10y(29) | 9.0 | Imprecise values supplied | <0.75mmol/L if < 2 years old <0.7mmol/L if ≥ 2 years old | 10.3 | 473 | 1.9 |
| Knijnenburg 2012(30) | 22.6 | 75/112 | males < 0.75 mmol/L; females < 0.71 mmol/L; < 15 years of age <0.68 mmol/L; OR those receiving supplementation | 12.1 | 320 | 5.9 |
| Pietilä 2005(34) | 71.4 | 14/14 | Not stated | 6.0 | 528 | 6.0 |
| Canpolat 1996(28) | Not stated | N/a | Not stated | 0.8 | 1050 | 14.0^a^ |
| ^a^ Age at enrolment in study (relapsed osteosarcoma) | | | | | | |

*Supplementary table 4: Summary table for hypomagnesaemia*

# Supplementary Table 5

|  | **N to have calcium data / N to receive cisplatin** | **Method for measuring calcium** | **Threshold for defining hypocalcaemia** | **Prevalence of hypocalacemia** | **Statistical analysis** |
| --- | --- | --- | --- | --- | --- |
| Arga 2015(23) | 33/33 | Serum | < 2.3mmol/L | 12.1% | No significant difference between population that received cisplatin and ifosfamide compared to those that received cisplatin alone |
| Brock 1991(25) | 21/40 | Serum | 2 standard deviations below age-specific mean values | 0.0% | Not performed |
| Brock 1992(26) | 15/30 | Serum | < 2.3mmol/L | 0.0% | Not performed |
| Skinner 2009(29) | N/a | Serum | Age-related reference ranges not otherwise specified | Not stated | Not performed |
| Pietilä 2005(34) | N/a | Plasma & serum ionised | Local laboratory reference values | Not stated | No significant difference between cisplatin and no cisplatin groups in plasma / serum ionised calcium values (p = 0.358 and 0.419, respectively) |

*Supplementary table 5: Summary table for hypocalcaemia*

*Supplementary table 5: Summary table for hypocalcaemia*

# Supplementary table 6

|  | **N to have potassium data / N to receive cisplatin** | **Method for measuring potassium** | **Threshold for defining hypokalaemia** | **Prevalence of hypokalaemia** | **Statistical analysis** |
| --- | --- | --- | --- | --- | --- |
| Arga 2015(23) | 33/33 | Serum | <3.5 mmol/L | 0.0% | No significant difference between population that received cisplatin and ifosfamide compared to those that received cisplatin alone |
| Ariceta 1997(24) | 18/22 | Not stated | Not stated | 5.5% | Not performed |
| Pietilä 2005(34) | 14/14 | Serum | Local laboratory reference values | 7.1% | Significantly lower serum potassium values in CCS who received cisplatin than those that didn’t (3.7mmol/L vs 3.8mmol/L, p=0.026)  No significant different in prevalence of hypokalaemia |

*Supplementary table 6: Summary table for hypokalaemia*

# Summary Table 7

|  | **N to have phosphate data / N to receive cisplatin** | **Method for measuring phosphate** | **Threshold for defining hypophos-phataemia** | **Prevalence of hypophos-phataemia** | **Statistical analysis** |
| --- | --- | --- | --- | --- | --- |
| Arga 2015(23) | 33/33 | Serum | 6–12 years 1mmol/L; 13–16 years 0.9mmol/L; >16 years 0.77mol/L | 12.1% | Increased prevalence of hypophosphataemia in CCS treated with cisplatin and ifosfamide, compared to cisplatin alone (p =0.012) |
| Pietilä 2005(34) | 14/14 | Plasma | Local laboratory reference values | 21.4% | Patients treated with cisplatin had significantly lower phosphate values (1.09mmol/L vs 1.32mmol/L, p= 0.013) and a significantly higher prevalence of hypophosphatemia (p=0.016). |
| Canpolat 1996(28) | N/a | Serum | Not stated | Not stated | No appropriate subgroup analysis |
| Knijnenburg 2012(30) | 71/112 | Serum | < 0.81 mmol/L for adult CCS; age-dependent values for children ; OR supplementation required | Not stated | No appropriate subgroup analysis |

|  | **N to have phosphate data / N to receive cisplatin** | **Method for measuring phosphate** | **Threshold for defining hypophos-phataemia** | **Prevalence of hypophos-phataemia** | **Statistical analysis** |
| --- | --- | --- | --- | --- | --- |
| Arga 2015(23) | 33/33 | Serum | 6–12 years 1mmol/L; 13–16 years 0.9mmol/L; >16 years 0.77mol/L | 12.1% | Increased prevalence of hypophosphataemia in CCS treated with cisplatin and ifosfamide, compared to cisplatin alone (p =0.012) |
| Pietilä 2005(34) | 14/14 | Plasma | Local laboratory reference values | 21.4% | Patients treated with cisplatin had significantly lower phosphate values (1.09mmol/L vs 1.32mmol/L, p= 0.013) and a significantly higher prevalence of hypophosphatemia (p=0.016). |
| Canpolat 1996(28) | N/a | Serum | Not stated | Not stated | No appropriate subgroup analysis |
| Knijnenburg 2012(30) | 71/112 | Serum | < 0.81 mmol/L for adult CCS; age-dependent values for children ; OR supplementation required | Not stated | No appropriate subgroup analysis |

*Supplementary table 7: Summary table for hypophosphataemia*

# Supplementary table 8

| **Study** | **N to have proteinuria data / N to receive cisplatin** | **Method of measuring proteinuria** | **Threshold for proteinuria** | **Prevalence** | **Statistics** |
| --- | --- | --- | --- | --- | --- |
| Dekkers 2013(27) | 39/51 | urinary albumin : creatine ratio | ≥3.5 mg/mmol Cr (women) and ≥2.5 mg/mmol Cr (men) | 17.9% | Multivariate analysis:  >450mg/m^2^: OR: 5.19; 95% CI 1.21; 22.21; P=0.03  =< 450mg/m^2^: OR 1.73; 95% CI 0.44-6.85, P=0.44 |
| Knijnenburg 2012(30) | 94/112 | Urine dipstick for albuminuria | ≥25 mg/dl | 13.8% | Multivariate risk factor analysis as independent risk factor for cumulative cisplatin dose: OR per 100mg/m^2^: 0.95; 95% CI 0.81– 1.12; p=0.54  Multivariate analysis as categorical risk factor with mutually exclusive treatment groups: OR 2.2; 95% CI 0.94– 5.14; p=0.070 |
| Green 2021(31) | N/a | Urine dipstick for proteinuria | ≥30mg/dL | No subgroup analysis | Univariate analysis as categorical variable: OR 1.39; 95% CI 0.81-2.38; p = 0.231  Univariate analysis per 100mg/m^2^: OR 1.05; 95% CI 0.93-1.19; p =0.409 |
| Pietila 2005(34) | 14/14 | urinary protein concentration and further 24 hour urine collection if >0.1g/L | > 100 mg/L | 7.1% | No different between CCS treated with or without cisplatin (p=1.000) |
| Latoch 2021(32) | N/a | Urinary albumin concentration | between 30 and 300 µg/mg | Not stated | No subgroup analysis |

*Supplementary table 8: Summary table for proteinuria*

# Supplementary table 9

| **Nephrotoxicity Grade** | **GFR (ml/min/1.73m^2^)** | **Magnesium if < 2 years of age (mmol/L)** | **Magnesium if ≥ 2 years of age (mmol/L)** |
| --- | --- | --- | --- |
| 0 | ≥90 | ≥0.75 | ≥0.70 |
| 1 | 60-89 | 0.60-0.74 | 0.55–0.69 |
| 2 | 40-59 | 0.5-0.59 | 0.45–0.54 |
| 3 | 20-39 | 0.40-0.49 and no symptoms | 0.35–0.44 |
| 4 | <20 | Tetany, convulsion or <0.40 | <0.35 |

*Supplementary table 9: Scoring criteria for composite nephrotoxicity scoring*

# Supplementary Table 10

| **Study** | **N to have composite nephrotoxicity data / N to receive cisplatin** | **Mild nephrotoxicity (Score 0-1)** | **Moderate nephrotoxicity (Score 2-3)** | **Severe nephrotoxicity (score ≥4)** |
| --- | --- | --- | --- | --- |
| Arga 2015(23) | 33/33 | 6/33 (18.2%) | 7/33 (21.3%) | 2/33 (6.1%) |
| Skinner 2009 CPL 1y(29) | Imprecise numbers given | Not stated | 7/32 (31.8%) | 1/21 (4.8%) |
| Skinner 2009 CPL 10y(29) | Imprecise numbers given | Not stated | 6/27 (22.2%) | 4/27 (14.8%) |
| Skinner 2009 CPL + CBP 1y(29) | Imprecise numbers given | Not stated | 2/11 (18.2%) | 0% |
| Skinner 2009 CPL + CBP 10y(29) | Imprecise numbers given | Not stated | 1/12 (8.3%) | 0% |

*Supplementary table 10: Prevalence for composite nephrotoxicity according to study population and grade of severity*

# Supplementary Table 11

| **Study** | **N to have blood pressure data / N to receive cisplatin** | **Method for measuring blood pressure** | **Threshold for defining hypertension** | **Prevalence** | **Median follow-up time (years)** |
| --- | --- | --- | --- | --- | --- |
| Arga 2015(23) | 33/33 | Not stated | Not stated | 0% | 4.66 |
| Dekkers 2013(27) | 40/55 | Electronic measurement not otherwise specified | systolic BP ≥140 mmHg, diastolic BP ≥90 mmHg and / or use of any antihypertensive medication | 20% | 18.3 |
| Skinner 2009 CPL 1y (29) | N/a | Not stated | As per “National High Blood Pressure Education Program Working Group on High Blood Pressure in Children and Adolescents: the fourth report on the diagnosis, evaluation and treatment of high blood pressure in children and adolescents.” | Not stated | 1.1 |
| Skinner 2009CPL 10y(29) | Imprecise numbers supplied | Not stated | As above | 4% | 10.3 |
| Skinner 2009 CPL + CBP 1y (29) |  | Not stated | As above | Not stated | 1.1 |
| Skinner 2009 CPL + CBP 10y (29) | 12/12 | Not stated | As above | 16% | 10.3 |
| Knijnenburg 2012(30) | 107/112 | Not described | a systolic >140 mmHg or diastolic > 90mmHg in adults; ≥95th percentile for sex, age, and height using height z-scores for Dutch children in paediatric survivors; or use of anti-hypertensive medication in either group. | 14% | 12.1 |
| Pietilä 2005(34) | 14/14 | Measurement of the right arm in a sitting position using an electronic oscillometric method with the cuff covering at least two thirds of the upper arm | systolic BP ≥140 mmHg and / or diastolic BP ≥90 mmHg in adults; If <18y/o: ≥95th percentile for sex, age, and height if <18 years old | 6/14 (43%) | 6.0 |
| Brock 1991(25) | N/a | Not stated | Age specific centiles in younger children, on basis of age and length in older children | Not stated | 2.5 |
| Green 2021(31) | N/a | Not stated | Not stated | Not stated | 23.2 |
| Latoch 2021(32) | N/a | 3 measurements at 2 minutes intervals using sphygmomanometry not otherwise specified | ≥95th percentile for age, sex, and height | Not stated | 8.4 |

*Supplementary table 11: Summary table for hypertension*

# Supplementary table 12

| **Study** | **N to have other nephrotoxicity data / N to receive cisplatin** | **Nephro-toxicity measure** | **Method-ology** | **Threshold** | **Prevalence** | **Statistics** |
| --- | --- | --- | --- | --- | --- | --- |
| Arga 2015(23) | 33/33 | Renal threshold for phosphate (Tmp/GFR) | 24 hour urine collection | 2–12 years: <1.03mmol/L  12–16 years: <0.91mmol/L  >16 years: < 0.85mmol/L | 45.5% | Median Tmp/GFR was significantly lower (P = 0.011). and incidence of reduced Tmp/GFR in CCS was significantly higher (p = 0.003) to receive cisplatin and ifosfamide, as opposed to cisplatin alone |
| Dekkers 2013(27) | N/a | urinary beta-2-micro-globulin : creatinine ratio | Urine sample not otherwise specified | ≥0.04 mg/mmol Cr | Not stated | No significance in multivariate analysis looking at risk of high and low dose cisplatin (>450 OR 0.52 95% CI 0.08; 3.29; p=0.49) (≤450mg/m^2^ OR 0.58, 95% CI 0.15; 2.26, P=0.43) |
| Latoch 2021(32) | 16/16 | KIM1/Cr | Serum creatinine and urinary KIM1 via commercial immuno-assays | - | - | No correlation between KIM1/CR and cisplatin in univariate regression analysis (cisplatin (coeff. 0.002, p = 0.719, 95% CI), no multivariate analysis carried out. |
| Latoch 2021(32) | 16/16 | NGAL/Cr | Serum creatinine and urinary NGAL via commercial immuno-assays | - | - | Multivariate linear regression analysis, correlation between cumulative doses of cisplatin and (Coefficient 0.108; standard error 0.048, p=0.041; 95% CI 0.005-0.211).  Spearman correlation between NGAL/CR and cumulative cisplatin dose:  (r = 0.534, p < 0.05).  No such correlations between KIM1/Cr and cisplatin dose. |
| Pietilä 2005(34) | 14/14 | Serum cystatin C | Fasted overnight blood sample | Not stated | Not stated | No significant difference between CCS who did and did not receive cisplatin (p= 0.765) |
| Pietilä 2005(34) | 14/14 | Glycosuria | Urine sample not otherwise specified | >0.05 g/L | 14.3% | No significant difference between CCS who did and did not receive cisplatin (p= 1.000) |
| Pietilä 2005(34) | 14/14 | Urinary alpha-1microglobulin | Nephelo-metric measure-ment | ≥8 mg/L | 21.4% | No significant difference between CCS who did and did not receive cisplatin (p= 0.055) |

# Supplementary figure 1

*Supplementary figure 1: Sensitivity analysis for pooled reduced GFR prevalence*

# Supplementary document 1
